# Vital Signs: Estimated Effects of a Coordinated Approach for Action to Reduce Antibiotic-Resistant Infections in Health Care Facilities — United States

**Published:** 2015-08-07

**Authors:** Rachel B. Slayton, Damon Toth, Bruce Y. Lee, Windy Tanner, Sarah M. Bartsch, Karim Khader, Kim Wong, Kevin Brown, James A. McKinnell, William Ray, Loren G. Miller, Michael Rubin, Diane S. Kim, Fred Adler, Chenghua Cao, Lacey Avery, Nathan T.B. Stone, Alexander Kallen, Matthew Samore, Susan S. Huang, Scott Fridkin, John A. Jernigan

**Affiliations:** 1 National Center for Emerging and Zoonotic Infectious Diseases; 2 VA Salt Lake City Health Care System and Division of Epidemiology, University of Utah; 3 Public Health Computational and Operations Research, Johns Hopkins Bloomberg School of Public Health; 4 Center for Simulation and Modeling, University of Pittsburgh; 5 Torrance Memorial Medical Center; 6 Infectious Disease Clinical Outcomes Research Unit, Los Angeles Biomedical Research Institute, Harbor-UCLA Medical Center; 7 Division of Infectious Diseases and Health Policy Research Institute, University of California Irvine Health School of Medicine; 8 Department of Mathematics, University of Utah; 9 Pittsburgh Super Computing Center

## Abstract

**Background:**

Treatments for health care–associated infections (HAIs) caused by antibiotic-resistant bacteria and *Clostridium difficile* are limited, and some patients have developed untreatable infections. Evidence-supported interventions are available, but coordinated approaches to interrupt the spread of HAIs could have a greater impact on reversing the increasing incidence of these infections than independent facility-based program efforts.

**Methods:**

Data from CDC’s National Healthcare Safety Network and Emerging Infections Program were analyzed to project the number of health care–associated infections from antibiotic-resistant bacteria or *C. difficile* both with and without a large scale national intervention that would include interrupting transmission and improved antibiotic stewardship. As an example, the impact of reducing transmission of one antibiotic-resistant infection (carbapenem-resistant *Enterobacteriaceae* [CRE]) on cumulative prevalence and number of HAI transmission events within interconnected groups of health care facilities was modeled using two distinct approaches, a large scale and a smaller scale health care network.

**Results:**

Immediate nationwide infection control and antibiotic stewardship interventions, over 5 years, could avert an estimated 619,000 HAIs resulting from CRE, multidrug-resistant *Pseudomonas aeruginosa*, invasive methicillin-resistant *Staphylococcus aureus* (MRSA), or *C. difficile*. Compared with independent efforts, a coordinated response to prevent CRE spread across a group of inter-connected health care facilities resulted in a cumulative 74% reduction in acquisitions over 5 years in a 10-facility network model, and 55% reduction over 15 years in a 102-facility network model.

**Conclusions:**

With effective action now, more than half a million antibiotic-resistant health care–associated infections could be prevented over 5 years. Models representing both large and small groups of interconnected health care facilities illustrate that a coordinated approach to interrupting transmission is more effective than historical independent facility-based efforts.

**Implications for Public Health:**

Public health–led coordinated prevention approaches have the potential to more completely address the emergence and dissemination of these antibiotic-resistant organisms and *C. difficile* than independent facility–based efforts.

## Introduction

With the continuing emergence of antibiotic resistance, treatments for bacterial infections are increasingly limited, and in some patients, effective treatment options do not exist. Antibiotics are a lifesaving medical tool, and antibiotic resistance undermines the ability to fight infectious diseases. CDC estimates that antibiotic-resistant bacteria cause 2 million illnesses and approximately 23,000 deaths each year in the United States (1). Infections caused by resistant pathogens have the potential to affect persons both in and out of health care settings. In addition, almost 250,000 persons each year require hospital care for *C. difficile* infections (CDIs), which are typically associated with antibiotic use (1). Despite success in preventing these infections at individual health care facilities (2,3), the continued spread of antibiotic resistant pathogens and *C. difficile* has outpaced the development of new therapies (1).

Historically, infection control interventions designed to prevent spread of *C. difficile* and antibiotic-resistant pathogens have been independently implemented by individual health care facilities, without clear coordination among other facilities in the community, which often care for the same patients. Although improvements within independent facilities are necessary, they might not be sufficient to reduce spread. These independent efforts do not account for the importance of inter-facility spread through movement of patients who are colonized or infected with these organisms, or the impact that one institution’s practices might have on the antibiotic resistance encountered by neighboring facilities (4–6). To date, even when fully implemented, this independent facility–based effort has not adequately controlled inter-facility spread of antibiotic-resistant pathogens (7). In addition to optimizing implementation of infection control in every facility, an inter-facility coordinated approach to interrupt spread, facilitated by local or state-based oversight, has the potential to more effectively reduce the overall prevalence of antibiotic-resistant infections across all health care facilities within a community. The impact of such coordinated responses can be estimated through mathematical modeling, and assessment of the expected benefits can inform the development and implementation of these programs.

## Methods

### Estimating infection incidence and deaths

Projections of infections and deaths in the United States during 2014–2019 were derived from data obtained through CDC’s National Healthcare Safety Network (NHSN) and Emerging Infections Program (EIP). Four particularly problematic health care–associated infections (HAIs) were included: CRE, multidrug-resistant *Pseudomonas aeruginosa*, invasive MRSA, and CDIs (1). To estimate the percentage of antibiotic-resistant HAIs over the next 5 years, logarithmic models for multidrug-resistant *P. aeruginosa* and CRE were generated from the annual percentage of resistant isolates from device and procedure–associated HAIs reported to NHSN during 2009–2013, and the percentage resistant by year was estimated through 2019. To obtain the annual number of infections, the 2011 national estimates of pathogen-specific HAIs were multiplied by the projected percentage resistant for each pathogen (8). Projections for invasive MRSA and CDI were derived from EIP national surveillance from 2005–2012 for MRSA and 2011 for CDI (3,9). Mortality rates from EIP data or published literature were applied to the projected number of infections to determine associated mortality (1). Estimated numbers of infections and deaths averted with the implementation of an immediate national intervention were based on published reports of national interventions in other countries, where interventions combining interrupting transmission with improved inpatient antibiotic prescribing resulted in roughly 30%–50% fewer infections over 5 years (reductions varied by pathogen) (10–12).

### Estimating effect of a coordinated approach in a network

Two independently developed and complementary agent-based mathematical simulation models were used to measure the impact of a coordinated approach to prevent the spread of antibiotic-resistant organisms within a group of health care facilities interconnected through patient sharing (i.e., a network), using CRE as a test case. These agent-based models are computer simulations that represent hospitalized patients as “agents” and track their dynamic interactions with other patients and CRE status throughout the health care system. The first model assessed the impact of the coordinated approach in a simulated network of 10 health care facilities consisting of four acute care hospitals (including one long-term acute care hospital), and six free-standing nursing homes serving adult patients. Transfer of patients between facilities was calibrated based on actual transfer data from the U.S. Department of Veterans Affairs, supplemented with state inpatient database data (11). The period used to measure the impact was 5 years. The second model assessed the impact of a coordinated approach in a larger region and used the Regional Healthcare Ecosystem Analyst (RHEA), a simulation based on data from the network of all 28 acute care hospitals (including five long-term acute care hospitals) and 74 free-standing nursing homes serving adult patients in Orange County, California. The RHEA model, originally developed to simulate MRSA transmission (13–16), was re-parameterized to simulate spread of CRE within this larger health care network, and the period used to measure impact was 15 years.

With each model, the spread of CRE was simulated under three hypothetical scenarios (1): infection control activity currently in common use (common approach/status quo, or baseline activity with no augmented intervention) (2), augmented efforts implemented independently at individual subsets of facilities (independent efforts), and (3) coordinated augmented approach across a health care network (coordinated approach). Baseline activity simulations assumed that facilities applied contact precautions only to colonized or infected patients identified through routine tests. The independent efforts allowed for up to 15% of hospitals to begin active detection (i.e., CRE surveillance cultures) and isolation of CRE-colonized patients after a predetermined number of patients had been identified through routine clinical tests at each individual hospital. The coordinated approach allowed for all health care facilities to share CRE test results with a central public health authority, which used that information to strategically target prevention activity across multiple facilities. Notification of patient status as CRE-colonized or CRE-infected to facilities receiving a patient upon inter-facility transfer varied by model, and increased in frequency from independent efforts to coordinated approaches.

Both models simulated the movement of patients within and between different health care facilities and transmission of CRE in a health care network based upon key parameter estimates that included inter-facility patient movement, the proportion of colonized patients recognized by routine clinical tests, and effectiveness of barrier precautions in preventing transmission. Models were parameterized based on published data or calibrated to published estimates of CRE incidence and prevalence at acute care hospitals, long-term acute care hospitals, and nursing homes in regions where CRE outbreaks have occurred. Mean values for number of acquisitions and cumulative prevalence were calculated from simulations.*

## Results

### Projected national incidence of infections and deaths from several resistant organisms

**FIGURE 1:**
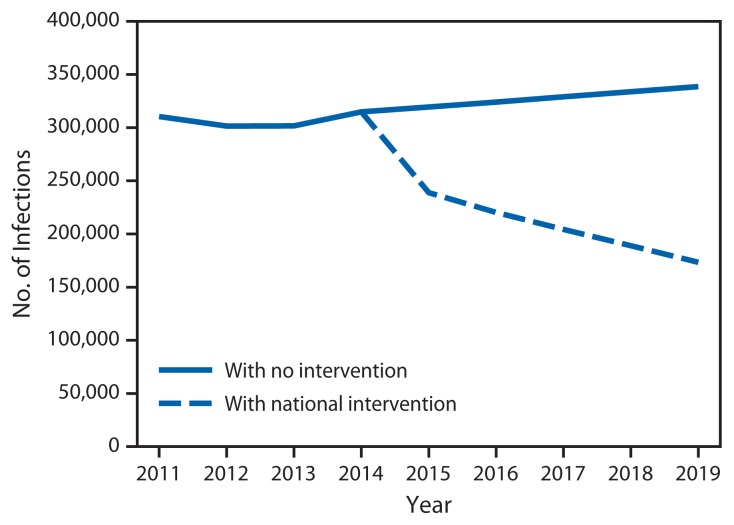
Comparison between the projected number of annual health care–associated infections from selected antibiotic-resistant bacteria* and *Clostridium difficile* with no intervention and the projected number with an aggressive national intervention — United States, 2014–2019^†^ * Methicillin-resistant *Staphlococcus aureus*, carbapenem-resistant *Enterobacteriaceae*, and multidrug-resistant *Pseudomonas aeruginosa*. ^†^ Additional information available at http://www.cdc.gov/drugresistance/resources/publications.html.

In 2011, an estimated 310,000 HAIs from CRE, multidrug-resistant *P. aeruginosa*, invasive MRSA, or CDI occurred in the United States. Based on current trends, in 5 years the number of infections caused by these pathogens is estimated to increase by approximately 10%, to 340,000 per year, unless additional interventions are implemented. With immediate implementation of national interventions combining infection control and antibiotic stewardship and, assuming similar effectiveness to that reported in other countries, an estimated 619,000 health care–associated infections and 37,000 deaths could be averted in 5 years (Figure 1).

### Estimated effect of coordinated approach in a network for reducing CRE spread

**FIGURE 2:**
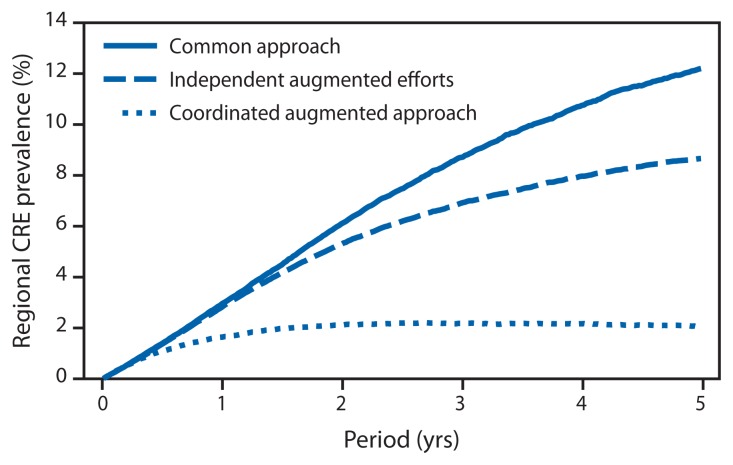
Projected regional prevalence of carbapenem-resistant *Enterobacteriaceae* (CRE) over a 5-year period under three different intervention scenarios — 10-facility model, United States* * Additional information available at http://www.cdc.gov/drugresistance/resources/publications.html. A video of the model simulations is available at http://www.cdc.gov/drugresistance/resources/videos.html.

For the 10-facility model, after the first introduction of CRE into the network, with baseline activity alone (no augmented intervention), the prevalence of health care–associated CRE infection or colonization after 5 years could be 12.2% with 2,141 patients acquiring CRE (Figure 2). With independent facility–augmented efforts, the prevalence of CRE after 5 years could be 8.6% with 1,590 patient acquisitions of CRE. Simulating a coordinated augmented approach, the model predicts a prevalence of 2.1% with 406 patient acquisitions after 5 years; the coordinated response resulted in a cumulative 81% reduction in CRE acquisitions, with 1,735 patient acquisitions prevented when compared with baseline activity (Figure 2) and a 74% reduction when compared with independent-facility efforts (Figure 2). On average, over this 5-year period, the coordinated approach resulted in 35 patients protected from CRE acquisition per 1,000 screening tests compared with 11 patients per 1,000 screening tests with the independent-facility efforts.†

**FIGURE 3:**
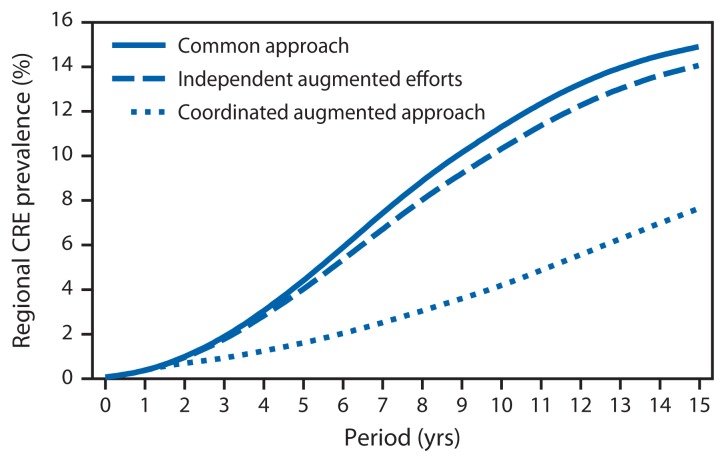
Projected countywide prevalence of carbapenem-resistant *Enterobacteriaceae* (CRE) over a 15-year period under three different intervention scenarios — 102-facility model, Orange County, California* * Additional information available at http://www.cdc.gov/drugresistance/resources/publications.html.

Using the 102-facility model of Orange County simulations over 15 years, the model estimated that the average network prevalence of CRE after 15 years would be 15% with 35,159 patients acquiring CRE (Figure 3). With independent facility-augmented efforts, the average network prevalence of CRE after 15 years could be 14% with 31,885 patient acquisitions of CRE. Simulating a coordinated approach in a network, the model predicted an average prevalence after 15 years of 8% with 12,614 patient acquisitions. Over 15 years, the coordinated response resulted in a cumulative 55% reduction in CRE prevalence with 19,271 patient acquisitions prevented, compared with independent-facility efforts.

## Conclusions and Comment

With effective action now, including nationwide antibiotic stewardship efforts and interventions to prevent spread of antibiotic-resistant infections, an estimated 619,000 infections caused by three problematic antibiotic-resistant HAIs or CDIs, and 37,000 deaths among infected patients might be averted nationally over the next 5 years. When considering published estimates of costs related to these four infections in the projections (17,18), an estimated $7.7 billion in direct medical costs could be averted (not including costs of implementing interventions). Optimizing implementation of basic infection control practice within individual facilities will be of fundamental importance to this effort. Further, models representing both large and small networks of interconnected health care facilities illustrate that a coordinated approach to interrupting transmission is more effective than traditional approaches that have relied on individual hospital efforts to independently identify and implement interventions. Incorporating such coordinated approaches at a national level could help ensure such actions are effective.

Several methods exist to coordinate prevention of antibiotic resistant HAIs; however, public health departments, particularly large local or state health departments, are uniquely suited to facilitate and accelerate this approach. Health departments are able to work with facilities within their jurisdiction in ways that amplify ongoing efforts of individual facilities or health systems. Because health departments possess substantial expertise in surveillance and prevention, they are well equipped to partner with multiple stakeholders, including hospitals, corporate and academic institutions, hospital associations, professional organizations, quality improvement organizations, and federal partners. Such state-based HAI antibiotic-resistance prevention programs can enable communities to locate the threat by sharing antibiotic resistance data and promoting accurate testing. Such programs also can respond quickly to prevent spread by identifying and rapidly responding to clusters, implementing a regionally coordinated response that includes opening lines of communication between facilities, helping individual hospitals improve infection prevention practices, and strategically targeting resources to prevent spread and maximize community impact.

***Key Points***

- Antibiotic use can cause germs to become resistant to antibiotics. Their use can also cause *Clostridium difficile* infections, which are quite contagious, especially in health care facilities.
- About 2 million illnesses and 23,000 deaths are caused by antibiotic resistant infections in the United States annually.
- About 250,000 people are hospitalized for *C. difficile* infections annually, typically caused by antibiotic use.
- If best infection control practices and antibiotic stewardship were nationally adopted, more than 600,000 infections and 37,000 deaths could be prevented over 5 years.
- If health care sites coordinated their patient infection information to guide interventions, an estimated 74% fewer patients would be infected by highly-resistant carbapenem-resistant Enterobacteriaceae over 5 years.
- Additional information is available at http://www.cdc.gov/vitalsigns.

Sharing the responsibilities to establish a coordinated program among communities of health care institutions with leadership by local health authorities will bring about the collective, shared benefits of coordination. Shifting the current culture to one of sharing information and sharing responsibility in prevention will require local leadership and commitment across various sectors. Developing a plan to share facility-level information regarding the presence and incidence of important antibiotic-resistant infections in ways that acknowledge the importance of protecting personally identifiable and other sensitive information, as occurred with facility-specific disclosure of HAI rates over the past decade, will be essential. Several key steps need to be taken to begin a coordinated approach. Health care facility leaders can take action to accelerate efforts to improve infection control practices within their own facilities and assure accurate and timely detection and reporting of antibiotic-resistant infections. In addition to augmented efforts, facilities can alert one another when enhanced infection control is needed for transferred patients who are colonized or infected with resistant organisms. Facility leadership should work with their respective health departments to determine best data sharing practices. Such steps improve access by public health departments to an established flow of HAI data, including those reported from hospitals to CDC’s NHSN. CDC is working to better assist health departments and health care facilities to collect, access, and respond to their HAI-related data, thereby enabling more efficient use of staff time and resources to implement effective prevention efforts.

A number of states have begun to develop programmatic capacity and experience in a coordinated approach for action to prevent antibiotic-resistant infections in health care settings. For example, the South Dakota Department of Health identified CRE in a region of the state, and in response, implemented a comprehensive program that included the introduction of mandatory reporting of CRE in 2013. The educational program was developed to increase CRE prevention knowledge among health care providers, and, with the two main hospital systems in the state, develop and implement interventions to reduce transmission. The program determines extent of spread and has worked with neighboring states to prevent cross-border transmission. This coordinated approach in oversight and rapid and efficient response resulted in a statewide decrease in CRE infections from 24 in 2012, to four in 2014. In Tennessee, the Department of Health has begun accessing data reported to NHSN and using analytic methods similar to The Targeted Assessment for Prevention§ strategy developed by CDC to target health care facilities with a disproportionate burden of CDI presenting to the hospital from the community or other facilities such as nursing homes. Such a strategy can identify gaps in infection prevention and antibiotic stewardship outside of hospitals. The Tennessee approach allows for prioritization of prevention efforts to the places where they will have their greatest impact. In Illinois, the Department of Public Health serves as a broker of data to all facilities in the state, maintaining a registry of patients infected or colonized with extensively drug-resistant bacteria. Currently, this registry is being used to report and identify patients with a history of CRE colonization or infection. Any registered facility can use the state-based notifiable disease reporting system to access the registry and determine if an anticipated admission involves a patient with a history of CRE. This allows appropriate infection control precautions to be taken at the time of admission.

The findings in this report are subject to at least five limitations. First, estimates of the projected number of infections and the impact of interventions are based on the assumption that rates will rise yearly according to current trends and that effective interventions will reduce annual rates of infections by 30%–50%. Second, reductions in infections with these four pathogens over the next 5 years might not translate into fewer HAIs overall; however, even if the infections prevented with these four pathogens are replaced by infections caused by less resistant organisms, such infections would be easier to treat. Third, the models were focused on interventions that are designed for interrupting transmission within and between health care facilities. Antibiotic resistant pathogens, such as MRSA, can also be spread in community settings; parallel efforts to prevent AR in the community are also of great importance. Fourth, illustration of the impact of coordinated approaches to preventing transmission as presented here is based on current understanding of CRE transmission within facilities and inter-facility transfer patterns, and some of the simplifying assumptions used in the simulations might bias the results. The use of Veterans Affairs data in the 10-facility model made it feasible to represent dependencies between lengths of stay, probabilities of readmission, and infection status. These are relationships that, in their basic form, likely are generalizable across health systems, and other models using different assumptions have suggested a similar advantage to regionally coordinated interventions involving other pathogens (4,5). Although the model assumptions incorporate active detection and isolation of CRE patients, the benefits illustrated in the model would be the same for any intervention (e.g., augmented hand hygiene efforts or skin antisepsis) that reduces transmission by the amount incorporated into the models. The analysis assumes no more than 15% of hospitals would implement augmented independent efforts. If a larger number of facilities implemented augmented independent efforts, the relative benefits of the coordinated approach would be lower, although as illustrated in the analysis, the resource utilization is much more efficient with coordination. Finally, the projected impact of interventions nationally include data for only four of the most problematic pathogens identified in the CDC Threat Assessment (1). These were chosen because they are propagated primarily in health care settings, are particularly difficult to treat, and have great potential to spread. Of note, the cost estimates assume the infections are not simply replaced with more susceptible bacteria and do not take into account the costs of implementing prevention programs, although a study on CDI prevention suggests such multifaceted prevention programs would be cost-saving (18).

The threat of antibiotic-resistant infections and CDI is not limited to certain areas or types of health care facilities. The current threat of antibiotic resistance in health care settings suggests that historical independent institution-based efforts to prevent transmission have been inadequate. Coordinated prevention approaches led by public health agencies, when coupled with intensified facility-based prevention programs, have the potential to more completely address the emergence and dissemination of these organisms.

## Footnotes

* Additional information available at http://www.cdc.gov/drugresistance/resources/publications.html.

† A video of the model simulations is available at http://www.cdc.gov/drugresistance/resources/videos.html.

§ Additional information available at http://www.cdc.gov/hai/prevent/tap.html.
